# Supplementary material for: An open-source framework for large-scale, flexible evaluation of biomedical text mining systems
Source: J Biomed Discov Collab. 2008 Jan 29;3:1. doi: 10.1186/1747-5333-3-1 (PMC2276192; doi:10.1186/1747-5333-3-1)
Supplement: Additional file 1 — Evaluation platform source code. The additional file contains the software evaluation framework discussed in this paper. The most current release can be downloaded from SourceForge.net [1]. See the accompanying README file for instructions on installation and use. Also included in the distribution is a collection of UIMA wrappers for some commonly used BioNLP tools and annotated corpora. [file 1747-5333-3-1-S1.zip › bionlp_uima_repository-1.0.1/docs/api/src-html/edu/uchsc/ccp/uima/mention/CCPMention.html]

```
001    
002    
003    /* First created by JCasGen Tue Aug 28 16:44:10 MDT 2007 */
004    package edu.uchsc.ccp.uima.mention;
005    
006    import org.apache.uima.jcas.JCas;
007    import org.apache.uima.jcas.JCasRegistry;
008    import org.apache.uima.jcas.cas.TOP;
009    import org.apache.uima.jcas.cas.TOP_Type;
010    
011    
012    /** The superclass for all CCP Mentions (class mention, complex slot mention, and non-complex slot mention)
013     * Updated by JCasGen Tue Aug 28 16:44:10 MDT 2007
014     * XML source: /home/williamb/eclipse/RELEASED_SOURCE_CODE/descriptors/typeSystem/CCPTypeSystem.xml
015     * @generated */
016    public class CCPMention extends TOP {
017      /** @generated
018       * @ordered 
019       */
020      public final static int typeIndexID = JCasRegistry.register(CCPMention.class);
021      /** @generated
022       * @ordered 
023       */
024      public final static int type = typeIndexID;
025      /** @generated  */
026      public              int getTypeIndexID() {return typeIndexID;}
027     
028      /** Never called.  Disable default constructor
029       * @generated */
030      protected CCPMention() {}
031        
032      /** Internal - constructor used by generator 
033       * @generated */
034      public CCPMention(int addr, TOP_Type type) {
035        super(addr, type);
036        readObject();
037      }
038      
039      /** @generated */
040      public CCPMention(JCas jcas) {
041        super(jcas);
042        readObject();   
043      } 
044    
045      /** <!-- begin-user-doc -->
046        * Write your own initialization here
047        * <!-- end-user-doc -->
048      @generated modifiable */
049      private void readObject() {}
050         
051     
052        
053      //*--------------*
054      //* Feature: mentionName
055    
056      /** getter for mentionName - gets The name of this mention.
057       * @generated */
058      public String getMentionName() {
059        if (CCPMention_Type.featOkTst && ((CCPMention_Type)jcasType).casFeat_mentionName == null)
060          jcasType.jcas.throwFeatMissing("mentionName", "edu.uchsc.ccp.uima.mention.CCPMention");
061        return jcasType.ll_cas.ll_getStringValue(addr, ((CCPMention_Type)jcasType).casFeatCode_mentionName);}
062        
063      /** setter for mentionName - sets The name of this mention. 
064       * @generated */
065      public void setMentionName(String v) {
066        if (CCPMention_Type.featOkTst && ((CCPMention_Type)jcasType).casFeat_mentionName == null)
067          jcasType.jcas.throwFeatMissing("mentionName", "edu.uchsc.ccp.uima.mention.CCPMention");
068        jcasType.ll_cas.ll_setStringValue(addr, ((CCPMention_Type)jcasType).casFeatCode_mentionName, v);}    
069      }
070    
071
```
